# Self-Assembled Nanotubes Based on Chiral H_8_-BINOL Modified with 1,2,3-Triazole to Recognize Bi^3+^ Efficiently by ICT Mechanism

**DOI:** 10.3390/mi15010163

**Published:** 2024-01-22

**Authors:** Jisheng Tao, Fang Guo, Yue Sun, Xiaoxia Sun, Yu Hu

**Affiliations:** 1 Jiangxi Key Laboratory of Organic Chemistry, Jiangxi Science and Technology Normal University, Nanchang 330013, China; taojisheng98@126.com (J.T.); guofang1025@163.com (F.G.); 2 State Key Laboratory of Molecular Engineering of Polymers, Shanghai Key Laboratory of Molecular Catalysis and Innovative Materials iChEM, Department of Chemistry, Fudan University, Shanghai 200433, China; sunyue19980307@163.com; 3 College of Chemistry, Nanchang University, Nanchang 330031, China

**Keywords:** self-assembled nanotubes, fluorescence sensors, Bi^3+^, intramolecular charge transfer effect (ICT), DFT calculation

## Abstract

A novel fluorescent “off” probe *R*-β-D-**1** containing a 1,2,3-triazole moiety was obtained by the Click reaction with azidoglucose using H_8_-BINOL as a substrate, and the structure was characterized by ^1^H NMR and ^13^C NMR and ESI-MS analysis. The fluorescence properties of *R*-β-D-**1** in methanol were investigated, and it was found that *R*-β-D-**1** could be selectively fluorescently quenched by Bi^3+^ in the recognition of 19 metal ions and basic cations. The recognition process of Bi^3+^ by *R*-β-D-**1** was also investigated by fluorescence spectroscopy, SEM, AFM, etc. The complex pattern of *R*-β-D-**1** with Bi^3+^ was determined by Job’s curve as 1 + 1, and the binding constant K_a_ of *R*-β-D-**1** and Bi^3+^ was valued by the Benesi–Hildebrand equation as 1.01 × 10^4^ M^−1^, indicating that the binding force of *R*-β-D-**1** and Bi^3+^ was medium. The lowest detection limit (LOD) of the self-assembled H_8_-BINOL derivative for Bi^3+^ was up to 0.065 µM. The mechanism for the recognition of Bi^3+^ by the sensor *R*-β-D-**1** may be the intramolecular charge transfer effect (ICT), which was attributed to the fact that the N-3 of the triazole readily serves as an electron acceptor while the incorporation of Bi^3+^ serves as an electron donor, and the two readily undergo coordination leading to the quenching of fluorescence. The recognition mechanism and recognition site could be verified by DFT calculation and CDD (Charge Density Difference).

## 1. Introduction

Supramolecular self-assembly is the formation of thermodynamically stable aggregates by molecular recognition or noncovalent bonding forces between basic structural units without human intervention. In these aggregates, the components also maintain their own intrinsic properties but exhibit certain overall characteristics due to their interactions and perturbations. These noncovalent interactions include hydrogen bonding, electrostatic interactions, π-π interaction, van der Waals forces and hydrophilic and hydrophobic forces [1]. It is well known that nature can assemble relatively simple molecular precursors into extremely complex biomolecules that are very important in living organisms, such as protein folding, nucleic acid assembly, phospholipid membranes and ribosomes. These self-assembly modalities have given researchers a lot of inspiration to obtain similar structures through these kinds of weak forces [2]. The properties of self-assembled materials change significantly compared to molecular materials [3]. As a cross-discipline between supramolecular chemistry and materials chemistry, the structural study of supramolecular self-assembly can help to enhance the understanding of the microstructure of molecular materials [4,5,6]. In the process of chemical development, molecular self-assembly nanomaterials have attracted more and more attention due to their wide applications in sensing, separation [7,8], catalysis [9,10], biomedicine [11,12,13], pharmacology [14,15,16] and other fields.

Bi^3+^ has a wide range of applications in various industries and fields. The presence of Bi^3+^ in a variety of materials and products is widely recognized, such as in the preparation and recovery of uranium, where it is used to absorb neutrons and stabilize the crystal lattice in nuclear fuel. This is critical in the field of nuclear energy to ensure the efficient and safe operation of nuclear power plants [17,18]. Studies have also shown that Bi^3+^ plays a crucial role in the production of semiconductor materials and alloys, particularly in the production of various photovoltaic cells [19,20,21], which are essential for the utilization of renewable energy sources. Bi^3+^ is also highly sought after in the cosmetic and pharmaceutical industries, where it is commonly incorporated into eye shadows, lipsticks and hair dyes to provide the desired aesthetic properties [22]. In the pharmaceutical industry, Bi^3+^ is used as an active pharmaceutical ingredient, particularly for the treatment of syphilis, peptic ulcers and hypertension [23,24]. It has been found that Bi^3+^ significantly improves the efficacy of various drugs, such as antibiotics and anti-inflammatory drugs. However, the benefits of Bi^3+^ are often outweighed by its potential risks if it is mishandled or used inappropriately. Identifying Bi^3+^ is therefore critical, especially when used in cosmetics and pharmaceuticals, where the presence of Bi^3+^ can have potentially harmful consequences, including causing diseases such as osteoarthritis and hepatitis, if not handled or used correctly. Therefore, the rapid and accurate identification of Bi^3+^ is necessary to prevent potential hazards and ensure consumer safety, and the development of sensitive and accurate Bi^3+^ detection methods is essential. Among the various assays that have been explored, fluorescent methods have been shown to be the most sensitive, accurate and efficient Bi^3+^ detection technique [25]. For example, when Wu et al. [26] studied the AIE effect based on glutathione-protected nonmetallic copper nanoparticles, the presence of Bi^3+^ effectively quenched the fluorescence of the sensor, thus achieving a faster and more sensitive fluorescence recognition of Bi^3+^. ShaJi et al. [27] studied the condensation reaction of toluenesulfonyl hydrazine and julolidine formaldehyde to obtain a novel dual-fluorescence colorimetric probe; in the presence of Bi^3+^, it achieved a visual color change and turn on fluorescence response to Bi^3+^ in human skeletal tumor cells through cellular imaging.

Since BINOL (binaphthol) is a unique organic compound with a C2 axisymmetric aromatic backbone, its conformation is exceptionally stable. This special structure gives it a rigid structure as well as good optical activity. In addition, its unique properties make it easy to modify, with many positions that can be chemically reacted to produce different derivatives. For example, the 3, 3′, 4, 4′ and 6, 6′ positions in the molecule can be modified by introducing different groups to realize various transformations. In addition, the two hydroxyl groups at its 2 position can be easily linked to other groups to synthesize a wide range of different compounds, thus expanding its potential for use and application. The simplicity and affordability of BINOL make it an ideal material for a wide range of applications, appealing to a wide variety of industries and scientific research. One of the most notable applications of BINOL is its use as a fluorescent probe for the rapid, sensitive recognition of metal ions and chiral amino acids [28,29]. This unique property stems from BINOL’s chiral photoactivity, which means that it fluoresces when irradiated with light at specific wavelengths. This makes it highly sensitive and capable of rapidly detecting trace amounts of metal ions or amino acids, making it an excellent tool for a variety of analytical applications [30]. Yu et al. [31] synthesized a small molecule gel based on BINOL phosphoric acid, which improved the gelation ability by complexing with Cu^2+^; *L*-histidine was highly sensitive to the formed organometallic gel and caused it to undergo gel collapse, while *D*-histidine did not affect it, enabling the enantiomeric recognition of histidine. Pu et al. [32] utilized the dynamic public chemistry of imines and found that the bis-aldehyde structure with BINOL as the substrate could undergo regioselective and enantioselective macrocyclization reactions with chiral diamines, whereas better enantioselectivity with Zn^2+^ coordination allowed high chemoselectivity and enantioselectivity for lysine.

In recent years, the synthesis, structural analysis and application of 1,2,3-triazole-modified sugar derivatives and sugar-based fluorescent chemosensors have received increasing attention because of their potential to greatly expand the fields of glycoscience and biotechnology. We investigated BINOL-glucose derivative fluorescent sensors, which exhibit high enantioselectivity and sensitivity to silver ions and are not interfered with by Hg^2+^ [33]. In addition, according to studies, triazoles can be used as corrosion inhibitors for organocopper [34]; especially, 1,2,4-triazole group derivatives are the most effective, whereas 1,2,3-triazole has a better affinity for metal ions, with an easier complexation with metal ions compared to 1,2,4-triazole [35,36]. On the other hand, 1,2,3-triazoles have a higher affinity for and are more readily coordinated to metal ions (especially Fe^3+^ and Cu^2+^), as confirmed by several studies [37,38]. However, few studies have investigated the complexation of 1,2,3-triazole structures with the metal Bi^3+^. Therefore, we introduced a glucose-modified triazole at position 3 of H_8_-BINOL and used the 1,2,3-triazole moiety and H_8_-BINOL as the recognition site and fluorophore, respectively. The aim of this study was to investigate monosubstituted glucose-modified triazole sensors that self-assembled into nanotubes in ethanol and into small spherical shapes in methanol. In addition, the study explored the application of this derivative in the molecular recognition of metal ions and found that it exhibited a unique fluorescence response to Bi^3+^, and DFT (density functional theory calculations) and CDD (Charge Density Difference) calculations were utilized to confirm the mechanism of the sensor’s recognition of Bi^3+^. 

## 2. Materials and Methods

To ensure a high purity and reproducibility of our experiments, all solvents were meticulously prepared and, if necessary, redistilled several times to remove any unwanted organic or inorganic impurities. We selected only analytical grade chemicals, which were either obtained directly from trusted pharmaceutical companies or synthesized through well-established controlled laboratory protocols. This approach ensured that the purity of our reagents was superior to most off-the-shelf products. To prepare the 0.1 M metal ion methanol solutions, we used the corresponding metal nitrates or chlorides. Unless otherwise stated in the experimental protocol, BiCl_3_ was the primary source of Bi^3+^, TMS was used as an internal standard and chloroform-D or dimethylsulfoxide-D_6_ was used as the solvent. We used a Germany Bruker AM-400WB spectrometer to measure the ^1^H NMR and ^13^C NMR spectra of the compounds and a Hitachi F-7100 fluorescence spectrometer from Hitachi High-Tech in Japan, to measure the fluorescence properties of the sensors, while high-resolution mass spectra were obtained by Germany Bruker Amazon SL ion trap MS tests.

In a 100 mL aubergine flask, G**1** (1-bromo-2,3,4,6-tetra-O-acetyl-α-D-glucose, 2.00 g, 4.86 mmol) was accurately weighed, the reaction system was sealed and TMSN_3_ (azidotrimethylsilane, 2.12 g, 18.48 mmol) and TBAF (tetrabutylammonium fluoride, 5.07 mL, 18.48 mmol) in 15 mL THF were added under the protection of argon gas. The reaction was carried out at room temperature for 24 h. The complete reaction of the raw materials was shown by TLC (V(ethyl acetate):V(petroleum ether) = 1:4), and the solvent was evaporated by rotary evaporator and then separated by column chromatography to obtain 1.31 g of white solid G**2** (1-azido-2,3,4,6-tetra-O-acetyl-β-D-glucose), which was obtained as a 72.1% yield. ^1^H NMR (400 MHz, DMSO-d_6_) δ 5.30 (t, J = 9.6 Hz, 1H), 5.11 (d, J = 8.9 Hz, 1H), 4.96 (t, J = 9.5 Hz, 1H), 4.80 (t, J = 9.3 Hz, 1H), 4.17–4.02 (m, 3H), 2.01 (d, J = 4.7 Hz, 12H).^13^C NMR (100 MHz, DMSO-d_6_) δ 170.10, 169.33, 169.20, 139.25, 86.39, 73.66, 72.93, 71.98, 70.41, 67.96, 67.16, 61.81, 60.60, 20.59.

*R-***P** (*R*-2′-(prop-2-yn-1-yloxy)-5,5′,6,6′,7,7′,8,8′-octahydro-[1,1′-binaphthalen]-2-ol, 0.20 g, 0.60 mmol) and G**2** (1-azido-2,3,4,6-tetra-O-acetyl-β-D-glucose, 0.38 g, 1.02 mmol). Under argon protection, 5 mL of tetrahydrofuran was added to the system. It was stirred in an ice bath for a few minutes to dissolve completely, then sodium ascorbate (0.25 g, 0.66 mmol) and anhydrous copper sulfate (0.17 g, 0.66 mmol) were accurately weighed, dissolved in 4 mL of deionized water and shaken well. When the mixed solution turned earthy yellow, it was added to the reaction system. The system was reacted for 10 h at room temperature for TLC (V(petroleum ether):V(ethyl acetate) = 4:1). The reaction was quenched by adding 5 mL of ice water to the reaction system and then extracted three times with 15 mL of dichloromethane. The extracted organic phases were then combined, washed with saturated NaCl solution and dried with MgSO_4_ anhydrous for 30 min. After filtration and rotary evaporation of the solvent, the separation was carried out by column chromatography (V(ethyl acetate):V(petroleum ether) = 1:1). About 0.32 g of white solid *R*-β-D-**1** was obtained as a 77.8% yield. MS-ESI *m*/*z* calcd for (C_37_H_43_N_3_O_11_+H^+^) = 706.2970, found 706.2980; ^1^H NMR (400 MHz, DMSO-d_6_) δ 8.55 (s, 1H), 7.98 (s, 1H), 6.88 (q, J = 8.4 Hz, 2H), 6.74 (d, J = 8.1 Hz, 1H), 6.58 (d, J = 8.2 Hz, 1H), 6.26 (d, J = 8.6 Hz, 1H), 5.57–5.40 (m, 2H), 5.09 (t, J = 9.6 Hz, 1H), 4.92 (q, J = 12.9 Hz, 2H), 4.40–4.22 (m, 1H), 4.15–3.93 (m, 2H), 2.73–2.52 (m, 4H), 2.33–1.85 (m, 12H), 1.84–1.32 (m, 12H). ^13^C NMR (100 MHz, DMSO-d_6_) δ 169.91, 169.45, 169.28, 169.03, 168.24, 153.09, 151.78, 144.42, 139.06, 136.17, 135.68, 129.65, 128.13, 126.81, 123.32, 122.76, 112.74, 111.58, 83.68, 73.47, 73.23, 72.05, 70.22, 67.53, 66.96, 65.27, 62.00, 61.78, 60.40, 28.76, 26.72, 26.59, 22.72, 22.63, 20.42, 20.31, 20.18, 19.74.

## 3. Results

**Scheme 1:**
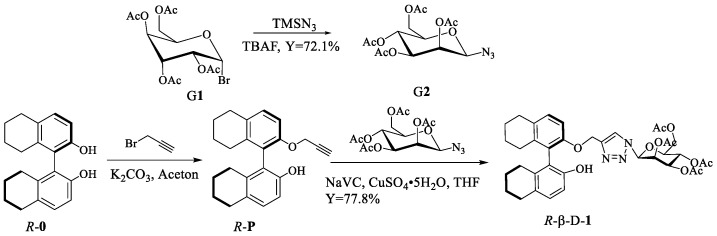
Synthesis route for the fluorescent probe *R-*β-D-**1**.

According to Scheme 1, the relatively easily synthesized *R*-**P** compound was synthesized from *R*-**0** (*R*-H_8_-BINOL) and 3-bromo-1-propyne, producing the compound as a yield of 69.6%. In contrast, azido glucose was obtained by introducing G**1** (1-bromo-2,3,4,6-tetra-O-acetyl-α-D-glucose) into the azide group with the azide reagent TMSN_3_ under TBAF catalysis to obtain G**2** (1-azido-2,3,4,6-tetra-O-acetyl-β-D-glucose). Then, the Click reaction between *R*-**P** and G**2** was catalyzed by Cu^+^ (formed by the reduction of copper sulfate with sodium ascorbate) to obtain the desired target sensor. The yield of this process was found to be 77.8%. The structure of the resulting *R*-β-D-**1** compound was characterized using ^1^H NMR, ^13^C NMR and ESI-MS.

### 3.1. Fluorescence Experiments of R-β-D-**1**

**Figure 1:**
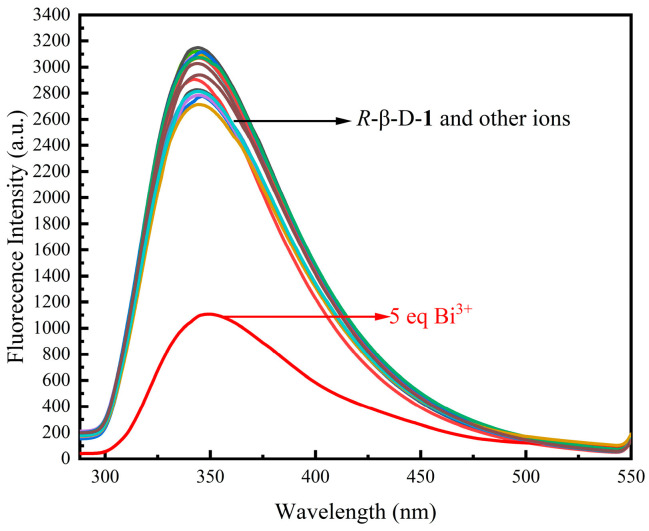
Fluorescence spectra of *R*-β-D-**1** (20 μM, MeOH solution) in the presence of different ions (Ba^2+^, Mn^2+^, Cu^+^, Ca^2+^, K^+^, Co^3+^, Cr^3+^, Zn^2+^, Al^3+^, Mg^2+^, Pb^2+^, Ag^+^, Cd^2+^, Gd^3+^, Li^+^, Na^+^, NH_4_^+^, Ni^2+^ and Bi^3+^) (*R*-β-D-**1** + 5-fold equivalent ions).

The fluorescent properties of *R*-β-D-**1** were next explored. It is shown in Figure 1 that *R*-β-D-**1** emits mid-intensity fluorescence at 335 nm (λ_ex_ = 278 nm, EX slit = 2.5 nm, EM slit = 2.5 nm). The effects of different cations (Ba^2+^, Mn^2+^, Cu^+^, Ca^2+^, K^+^, Co^3+^, Cr^3+^, Zn^2+^, Al^3+^, Mg^2+^, Pb^2+^, Ag^+^, Cd^2+^, Gd^3+^, Li^+^, Na^+^, NH_4_^+^, Ni^2+^ and Bi^3+^) on *R*-β-D-**1** in methanol solution were investigated by fluorescence spectrophotometry. The fluorescence of the sensor *R*-β-D-**1** showed significant fluorescence quenching in response to Bi^3+^, whereas it did not show significant fluorescence quenching for other ions under the same conditions. This demonstrates that *R*-β-D-**1** is capable of the specific fluorescence recognition of Bi^3+^.

### 3.2. Ion Competition Studies

**Figure 2:**
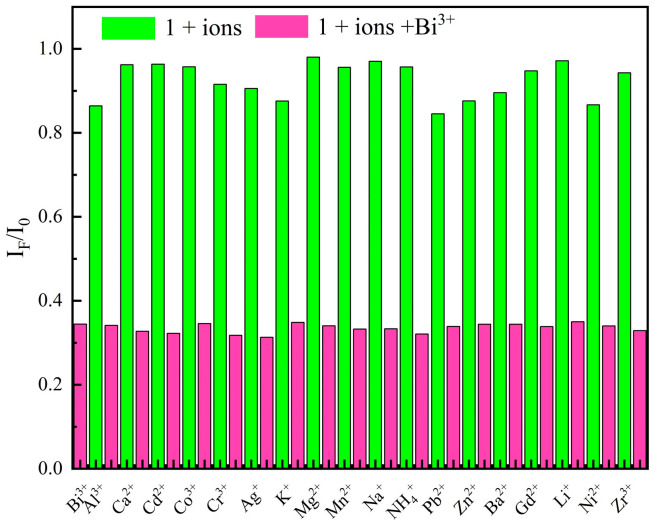
Green bars in the graph indicate the addition of 5.0 eq of different cations to the *R*-β-D-**1** (20 μM in CH_3_OH) solution; magenta bars in the graph indicate the addition of competing cations in the presence of Bi^3+^. I_F_/I_0_ indicates the degree of fluorescence quenching. Where I_0_ denotes the intensity of fluorescence of *R*-β-D-**1** only, and I_F_ denotes the intensity of fluorescence of a mixture of competing metal ions and Bi^3+^.

To further investigate the selectivity and specificity of the fluorescence detection of Bi^3+^ by *R*-β-D-**1**, we performed an ion competition experiment for *R*-β-D-**1** using fluorescence emission spectroscopy, as shown in Figure 2. Other 5.0 equivalents of cations and the same equivalents of Bi^3+^ were added to the *R*-β-D-**1** detection solution, and the fluorescence intensity was measured at λ_ex_ = 278 nm. The results showed that other metal ions had little effect on *R*-β-D-**1**-Bi^3+^. Meanwhile, we excluded the effect of the presence of anions in the solvent when we added to the *R*-β-D-**1** assay solution after adding 5.0 eq of Bi^3+^, to which we added equal amounts of different anions (NO_3_^−^, Cl^−^, CN^−^, SO_4_^2−^, SO_3_^2−^, CO_3_^2−^, HCO_3_^−^, SiO_3_^2−^, HSO_4_^−^, HSO_3_^−^, H_2_PO_4_^−^, ClO_2_^−^, S_2_O_3_^2−^, HPO_4_^2−^, C_2_O_4_^2−^ and CH_3_COO^−^ cations were K^+^ or Na^+^). Fluorescence detection at λ_ex_ = 278 nm yielded a fluorescence detection histogram (Figure S8 in the Supplementary Materials), and it could be found that these anions had little effect on the process of *R*-β-D-**1** recognition with Bi^3+^.This demonstrated that *R*-β-D-**1** could produce a selective fluorescence response to Bi^3+^ in the presence of background competing ions, and thus, *R*-β-D-**1** could be used for specific fluorescent sensors.

**Figure 3:**
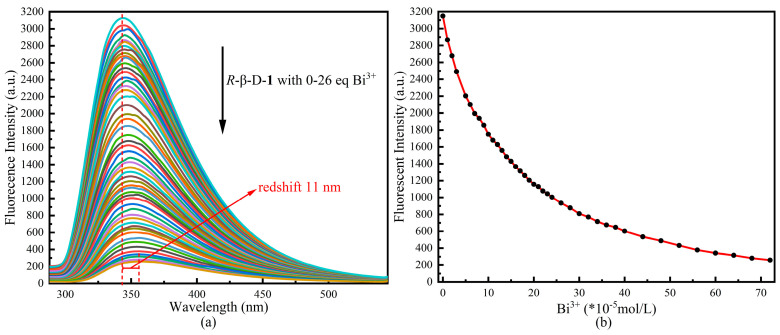
(**a**) Fluorescence titration plot of sensor *R*-β-D-**1** (20 μM in CH_3_OH, λ_ex_ = 278 nm) versus Bi^3+^ (0.01 M) equivalents (0–26.0 eq). (**b**). Plot of the change in fluorescence intensity of sensor *R*-β-D-**1** at 343 nm as the concentration of Bi^3+^ increases.

The results of the concentration-dependent fluorescence experiments of the sensor *R*-β-D-**1** on Bi^3+^ are shown in Figure 3. According to the results of the titration experiment, the fluorescence intensity of *R*-β-D-**1** at 343 nm was gradually diminished and red-shifted to 354 nm when the concentration of Bi^3+^ was increased from 0-fold equivalent to 26-fold equivalent, as shown in Figure 3a. The fluorescence intensity of *R*-β-D-**1** decreased to the minimum with the increase of the concentration of Bi^3+^, and the maximal fluorescence intensity showed a good linear relationship with the concentration of Bi^3+^, as shown in Figure 3b.

**Figure 4:**
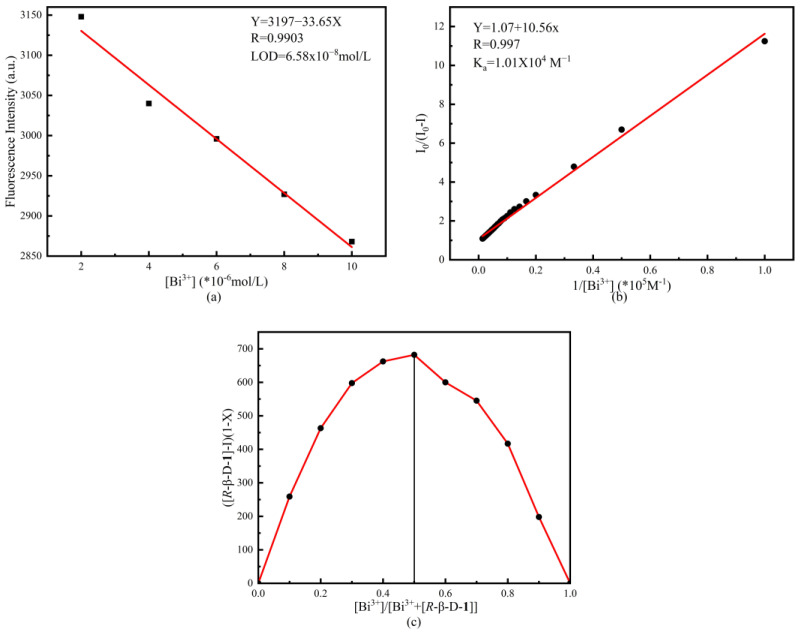
(**a**) The lowest limit of detection (LOD) of sensor *R*-β-D-**1** for Bi^3+^ was 6.58 × 10^−8^mol/L. (**b**) A 1:1 combination based on the graphs of stoichiometric I_0_/(I_0_-I) and 1/[Bi^3+^] Benesi–Hildebrand equations. (**c**) Working curve for the 1:1 compounds of *R*-β-D-**1** with Bi^3+^, where x is the molarity of Bi^3+^.

In order to determine the complexation of *R*-β-D-**1** with Bi^3+^, we investigated the detection limits and complexation constants of *R*-β-D-**1** with Bi^3+^ and then plotted Job’s plots using the reported method. Based on the concentration-dependent fluorescence titration experiment (Figure 4a), the minimum limit of detection (LOD) of the novel sensor *R*-β-D-**1** for Bi^3+^, 0.065 µM, was calculated based on “*LOD* = *3σ*/*S*” (σ is the signal standard deviation of the fluorescence intensity of nine blank samples of the sensor, while S is the slope of the concentration-dependent experiment). The K_a_ of the novel sensor *R*-β-D-**1** for Bi^3+^ was calculated based on the values of the I_0_/(I_0_-I) and 1/[Bi^3+^] plots, and the complexation constant of *R*-β-D-**1** with Bi^3+^ was calculated using the Benesi–Hildebrand equation to be 1.01 × 10^4^ M^−1^, as shown in Figure 4b. The total concentration of the mixed solution of *R*-β-D-**1** and Bi^3+^ was kept at 2.0 × 10^−5^ M(R = 0.990) during the test. The results in Figure 4c indicate that, when [Bi^3+^]/([*R*-β-D-**1**]+[Bi^3+^]) was about 0.5, the molar fraction of [Bi^3+^] reached a maximum value, indicating that the sensor *R*-β-D-**1** formed a 1 + 1 complex with Bi^3+^.

### 3.3. SEM Microscopy Study

**Figure 5:**
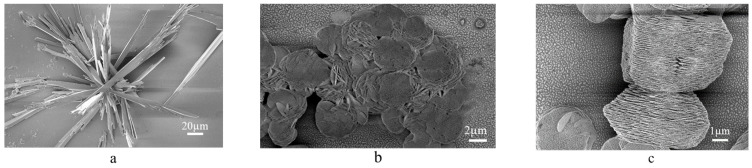
(**a**) SEM image of *R*-β-D-**1**. (**b**,**c**) SEM images of different angles in the presence of Bi^3+^.

In order to investigate the recognition process of *R*-β-D-**1** and Bi^3+^, the sensor was dissolved in ethanol and dispersed by ultrasonic dispersion and used for SEM inspection to obtain Figure 5. As shown in Figure 5a, it can be seen that *R*-β-D-**1** self-assembled together to form a micrometer-sized particle with a hollow structure in the middle of the particle; after the addition of Bi^3+^, the structure on the surface of the particle formed a flower-like structure (Figure 5b) induced by Bi^3+^, and the particle had a certain height, as shown in Figure 5c, and it was formed by many lamellar structures stacked together. Since our fluorescence test was performed in methanol solvent, in order to exclude the effect of solvent polarity, we wanted to know whether the self-assembly effect of the sensor in methanol solvent and the morphology after recognition with Bi^3+^ was related to the effect by the polarity of an ethanol solvent. Therefore, we observed the self-assembly of the fluorescent sensor *R*-β-D-**1** in methanol solution. *R*-β-D-**1** was dissolved in methanol and dispersed by ultrasonication followed by SEM inspection, and the morphology was obtained as shown in Figure S9a in the Supplementary Materials, which was found to be different from that in ethanol, self-assembling into a small ball-mounted structure, suggesting that the polarity of the solvent had a large effect on the self-assembly of the sensor *R*-β-D-**1**. However, after the addition of Bi^3+^, it could be found through Figure S9b that, similar to the recognition effect in ethanol, they both formed flower-like morphology, indicating that the change in the polarity of the solvent (methanol or ethanol) does not affect the recognition effect of sensor *R*-β-D-**1** with Bi^3+^.

**Figure 6:**
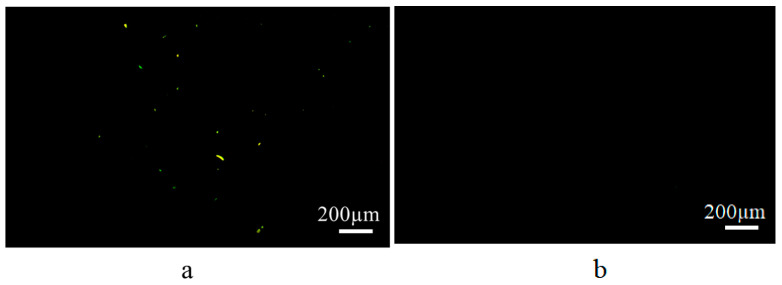
(**a**) AFM plot of sensor *R*-β-D-**1**, and (**b**) AFM plot after the addition of Bi^3+^ in *R*-β-D-**1**.

The recognition process of the sensor *R*-β-D-**1** with Bi^3+^ was observed by AFM (Figure 6). After dissolving *R*-β-D-**1** in ethanol and observing it under the microscope, the presence of many fluorescent particles could be seen, but the fluorescence disappeared after the addition of Bi^3+^, which was the same phenomenon as the fluorescence test.

### 3.4. A Plausible Sensing Mechanism for R-β-D-**1**

**Figure 7:**
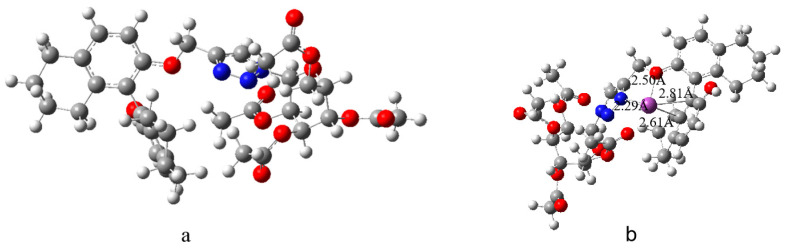
*R*-β-D-**1** structure of the sensor that was computationally optimized by density functional theory at the B3LYP/6-31G level of theoretical sophistication. (**a**) is the *R*-β-D-**1** structure of the sensor, (**b**) is the structure of the sensor after complexation of *R*-β-D-**1** with Bi^3+^.

Density functional theory calculations were performed to further understand the bonding nature of the sensor *R*-β-D-**1** with Bi^3+^. The density functional of B3LYP/6-31G was carried out using the program Gaussian 09W, and the dispersion correction of GD3BJ was taken into account to calculate the structure and energy levels of the sensor *R*-β-D-**1** before and after coordination with Bi^3+^ [39]. The structure of the sensor *R*-β-D-**1** after optimization is shown in Figure 7a. In the optimized structure of the complex of sensor *R*-β-D-**1**-Bi^3+^ (Figure 7b), Bi^3+^ is coordinated to the 3N atom of the 1,4-disubstituted triazole group to form a Bi^3+^-N double bond with a distance of 2.29 Å, as well as to the O atom attached to the benzene ring to form a Bi^3+^-O distance of 2.5 Å. In addition to this, Bi^3+^ formed Bi^3+^-C bonds with C atoms at 1′ and 2′ on the other benzene ring with bond lengths of 2.61 Å and 2.81 Å. The prerequisite for the formation of coordination bonds by the sensor *R*-β-D-**1**-Bi^3+^ was satisfied.

**Figure 8:**
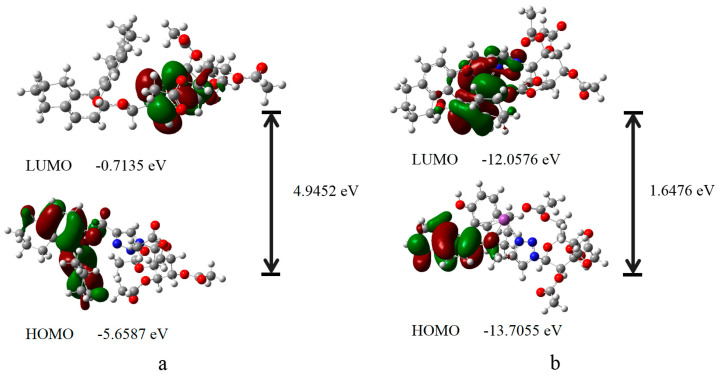
Molecular orbitals at the frontier of the sensor *R*-β-D-**1** optimized at the B3LYP/6-31G level of theory and structure after complexation with Bi^3+^. (**a**) is the HOMO-LUMO gap of the sensor *R*-β-D-**1**, (**b**) is the HOMO-LUMO gap of the sensor’s *R*-β-D-**1** complexed with Bi^3+^.

In addition, further observation of the electron cloud distribution of *R*-β-D-**1** (Figure 8) revealed that, in the absence of complexation with metal ions, the electron cloud was mainly located in the LUMO orbitals of the 1,2,3-triazole moiety and the HOMO orbitals of the benzene ring of H_8_-BINOL (Figure 8a) and that, once combined with Bi^3+^ (Figure 8b), the electron cloud was mainly concentrated in the bulky ring in which -OH was located in the LUMO orbitals in the benzene ring where -OH was located and in the HOMO orbitals in the benzene ring where methoxy was located, and all the band gaps (HOMO-LUMO Orbital Energy Difference) between the ground state energy and the excited state energy were reduced after Bi^3+^ complexation from the original 4.9452 eV to 1.6476 eV, suggesting that the existence of the interactions between the Bi^3+^ and *R*-β-D-**1** interaction induced fluorescence quenching [40,41,42].

**Figure 9:**
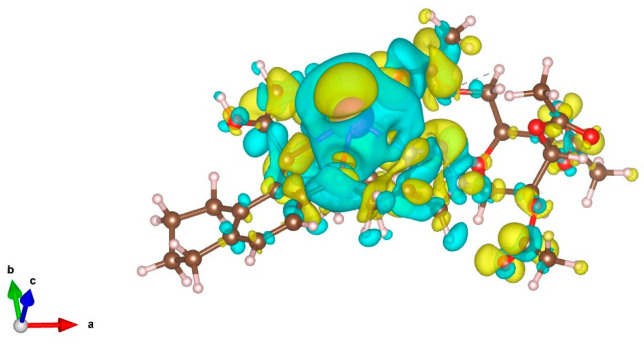
Differential charge density distributions of *R*-β-D-**1**-Bi^3+^. The yellow and blue colours represent electron accumulation and depletion regions, respectively, and the isosurface value is 0.001 e/Å3 (test methods are in the Supplementary Materials).

In addition, we also performed a differential charge density analysis using VASP to obtain Figure 9, where yellow and blue colors represent the electron accumulation and depletion regions, and it can be found that the charge of the 3-N of the 1,2,3-triazole moiety is shifted toward Bi^3+^ while the charge on Bi^3+^ is shifted toward the surrounding C atoms, which proves the existence of the ICT effect between *R*-β-D-**1** and Bi^3+^.

**Figure 10:**
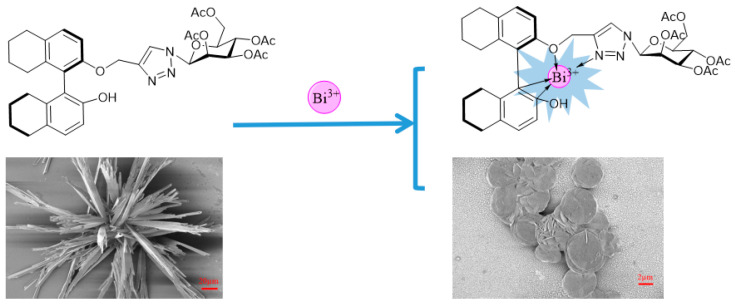
Mechanism of Bi^3+^ recognition by sensor *R*-β-D-**1**.

In summary, the plausible mechanism for the recognition of Bi^3+^ by the sensor *R*-β-D-**1** is the intramolecular charge transfer effect (ICT) [43,44], as shown in Figure 10, which is attributed to the fact that the N-3 of the 1,4-disubstituted triazole readily serves as an electron acceptor [45,46,47], whereas the incorporation of Bi^3+^ serves as an electron donor, and the two readily undergo coordination leading to the quenching of fluorescence.

## 4. Conclusions

A monosubstituted H_8_-BINOL glucose derivative was synthesized from azido glucose and *R*-H_8_-BINOL and characterized by ^1^H NMR, ^13^C NMR and MS. Its fluorescence properties in the presence of metal ions were investigated by fluorescence spectroscopy. The high selectivity and sensitivity of the *R*-β-D-**1** sensor in Bi^3+^ sensing were confirmed by fluorescence response experiments, competition experiments and fluorescence titration experiments on 19 different metal ions. The morphological changes of the sensor *R*-β-D-**1** before and after the addition of Bi^3+^ in different solvents were also investigated by scanning electron microscopy (SEM), and the stoichiometry and coordination of the complexes were determined by fluorescence emission spectroscopy, which confirmed the 1 + 1 binding mode between the sensor *R*-β-D-**1** and Bi^3+^, and the limit of detection (LOD) of the sensor *R*-β-D-**1** for Bi^3+^ was calculated to be 0.065 µM. In addition, the bonding situation of the sensor *R*-β-D-**1** was investigated using DFT and CDD to explore the recognition mechanism of the sensor *R*-β-D-**1** with Bi^3+^. In this paper, an “off” fluorescent probe was proposed for recognizing the transition metal Bi^3+^ with high selectivity and sensitivity.

## Data Availability

Data are contained within the article and Supplementary Materials.

## Supplementary Materials

The following supporting information can be downloaded at: https://www.mdpi.com/article/10.3390/mi15010163/s1, Figure S1. ^1^H NMR of 1-azido-2,3,4,6-tetra-O-acetyl-β-D-glucose (DMSO-d_6_). Figure S2. ^13^C NMR of 1-azido-2,3,4,6-tetraFi-O-acetyl-β-D-glucose (DMSO-d_6_). Figure S3. ^1^H NMR of *R*-**P** (DMSO-d_6_). Figure S4. ^13^C NMR of *R*-**P** (DMSO-d_6_). Figure S5. ^1^H NMR of *R*-β-D-**1** (DMSO-d_6_). Figure S6. ^13^C NMR of *R*-β-D-**1** (DMSO-d_6_). Figure S7. mass spectrometry of *R*-β-D-**1**. Figure S8. Fluorescence histogram of *R*-β-D-**1** versus Bi^3+^ in the presence of different anions. Figure S9. (a) SEM image of *R*-β-D-**1** in methanol solvent, (b) is the SEM morphology change of *R*-β-D-**1** with the addition of Bi^3+^. Refs. [48,49,50,51,52,53,54,55,56,57,58,59] are cited in the Supplementary Materials.
