# Supplementary material for: Self-Assembled Nanotubes Based on Chiral H8-BINOL Modified with 1,2,3-Triazole to Recognize Bi3+ Efficiently by ICT Mechanism
Source: Micromachines (Basel). 2024 Jan 22;15(1):163. doi: 10.3390/mi15010163 (PMC10821062; doi:10.3390/mi15010163)
Supplement: Supplementary file 1 [file micromachines-15-00163-s001.zip › micromachines-2775526-supplementary.pdf]

Supplementary materials

Self-Assembled Nanotubes Based on Chiral H_8-BINOL Modified with 1,2,3-Triazole to Recognize Bi^{3+} Efficiently by ICT Mechanism

Jisheng Tao [1], Fang Guo [1], Yue Sun[2], Xiaoxia Sun [1,*] and Yu Hu [3,*]

[1] Jiangxi Key Laboratory of Organic Chemistry, Jiangxi Science and Technology Normal University, Nanchang 330013, China.
[2] Department of Chemistry, State Key Laboratory of Molecular Engineering of Polymers, Shanghai Key Laboratory of Molecular Catalysis and Innovative Materials iChEM, Fudan University, Shanghai 200433, China.
[3] College of Chemistry, Nanchang University, Nanchang 330031, China.
Email: sunxiaoxia77@126.com (X. Sun). huyu@ncu.edu.cn (Y. Hu)

content

Content

Supplementary materials..1
1.Synthesis of *R*-P..2
2. ^{1}HNMR, ^{13}CNMR of 1-azido-2,3,4,6-tetra-O-acetyl-β-D-glucose (DMSO-D_6)..3
3. ^{1}HNMR, ^{13}CNMR of *R*-P (DMSO-D_6)..4
4. ^{1}H NMR,^{13}C NMR of *R*-β-D-1..5
5. MS plot of *R*-β-D-1..6
6.Histogram of *R*-β-D-1 with Bi^{3+} in the presence of different anions..6
7.Preparation of fluorescence test samples..6
8.Method of calculating charge density difference (CDD)..7
9.SEM plots of the sensor *R*-β-D-1 with Bi^{3+} in methanol..7

1.Synthesis of *R*-P

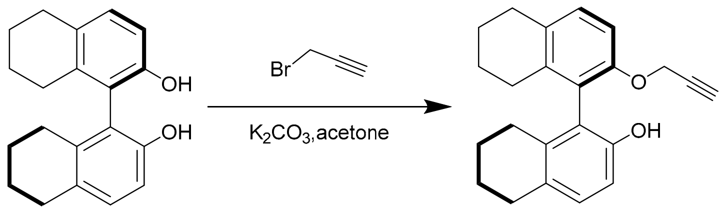

R-H_8-BINOL (2.0 g, 6.79 mmoL), potassium carbonate (1.88 g, 13.6 mmoL) and acetone (15.0 mL) as solvent were added to a 100 mL three-necked flask at room temperature. 3-Bromopropargyl (0.94 mL, 10.87 mmoL) was slowly added to the three-necked flask and stirred at room temperature for 5-7 min, heated to 60°C and refluxed for 12 h. The solution was then analyzed by TLC. The reaction was quenched when TLC detection (EA:PE=1:4) showed complete reaction of the raw material. The system was cooled to room temperature, the reaction liquid was filtered through a recirculating pump and the filtrate was washed three times with acetone. About 200-300 mesh of silica was added to the filtrate and the solvent was dried on a rotary evaporator to give a yellow-brown powder. Column chromatography using petroleum ether and ethyl acetate as eluents (V(PE):V(EA) = 15:1) yielded 1.56 g of yellow-brown solid in 69.3% yield.^{1}HNMR (400 MHz, DMSO-d_6) δ 8.73 (s, 1H), 7.13 (d, J = 8.4 Hz, 1H), 7.02 (d, J = 8.4 Hz, 1H), 6.95 (d, J = 8.2 Hz, 1H), 6.77 (d, J = 8.2 Hz, 1H), 4.73 (d, J = 2.5 Hz, 2H), 3.57 (s, 1H), 2.88 – 2.73 (m, 4H), 2.73 – 2.64 (m, 4H), 2.45 – 2.26 (m, 2H), 2.09 (dd, J = 30.4, 17.0 Hz, 2H).^{13}C NMR (101 MHz, DMSO-d_6) δ 152.02, 151.58, 135.90, 135.24, 129.30, 127.99, 127.74, 126.55, 126.15, 122.86, 112.50, 110.30, 79.69, 77.18, 55.08, 28.51, 26.48, 26.35, 22.55, 22.45.

2. ^{1}HNMR, ^{13}CNMR of 1-azido-2,3,4,6-tetra-O-acetyl-β-D-glucose (DMSO-D_6)

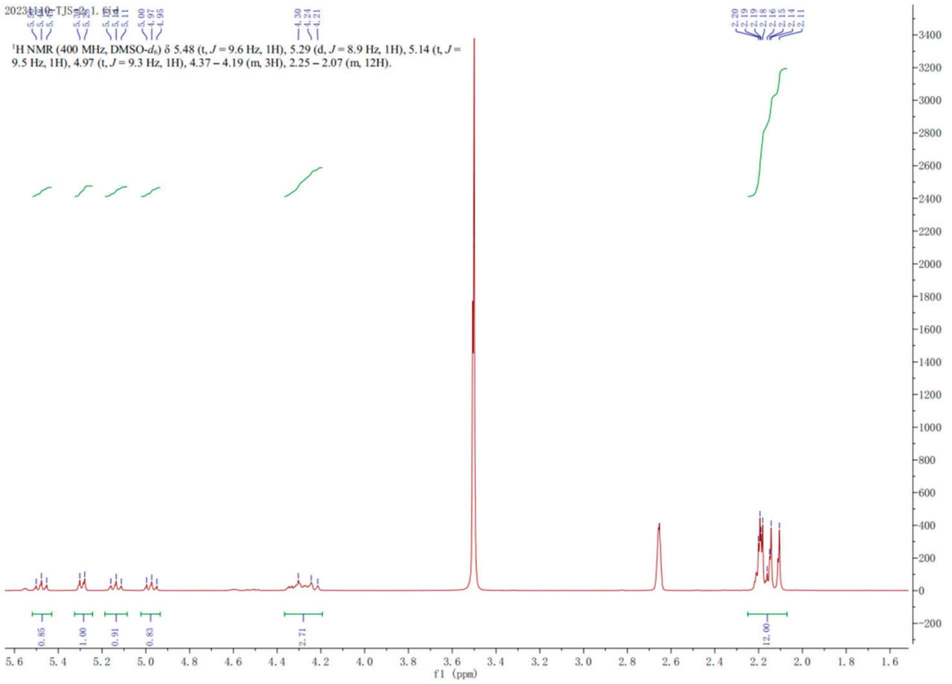

Figure S1.^{1}H NMR of 1-azido-2,3,4,6-tetra-O-acetyl-β-D-glucose (DMSO-d_6)

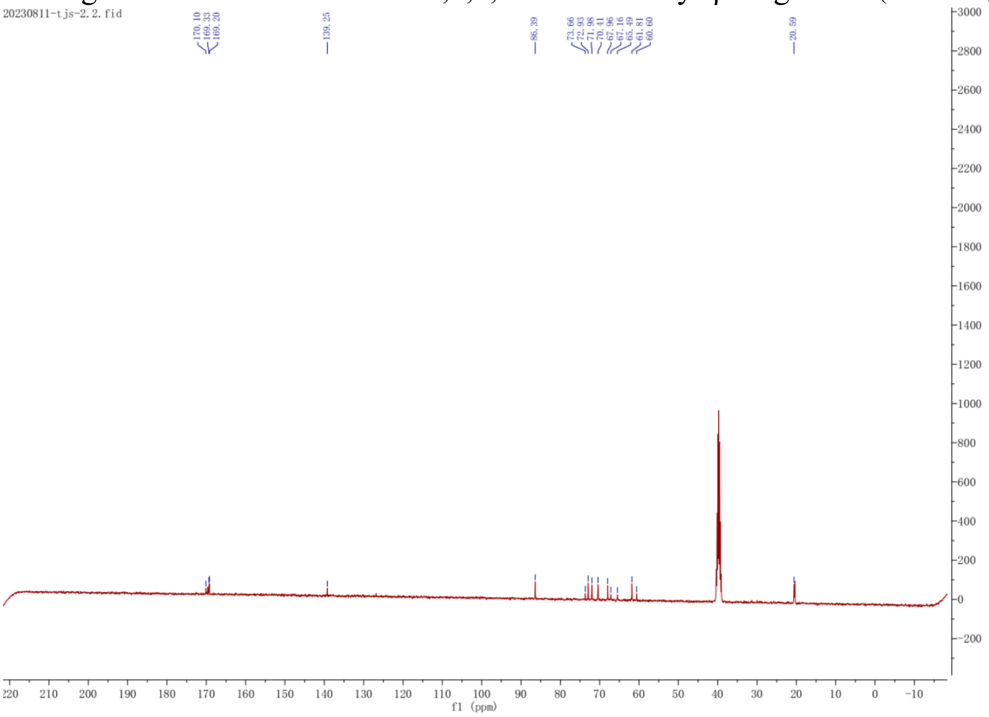

Figure S2 ^{13}C NMR of 1-azido-2,3,4,6-tetra-O-acetyl-β-D-glucose (DMSO-d_6)

3. ^{1}HNMR, ^{13}CNMR of *R*-**P** (DMSO-D$_6$)

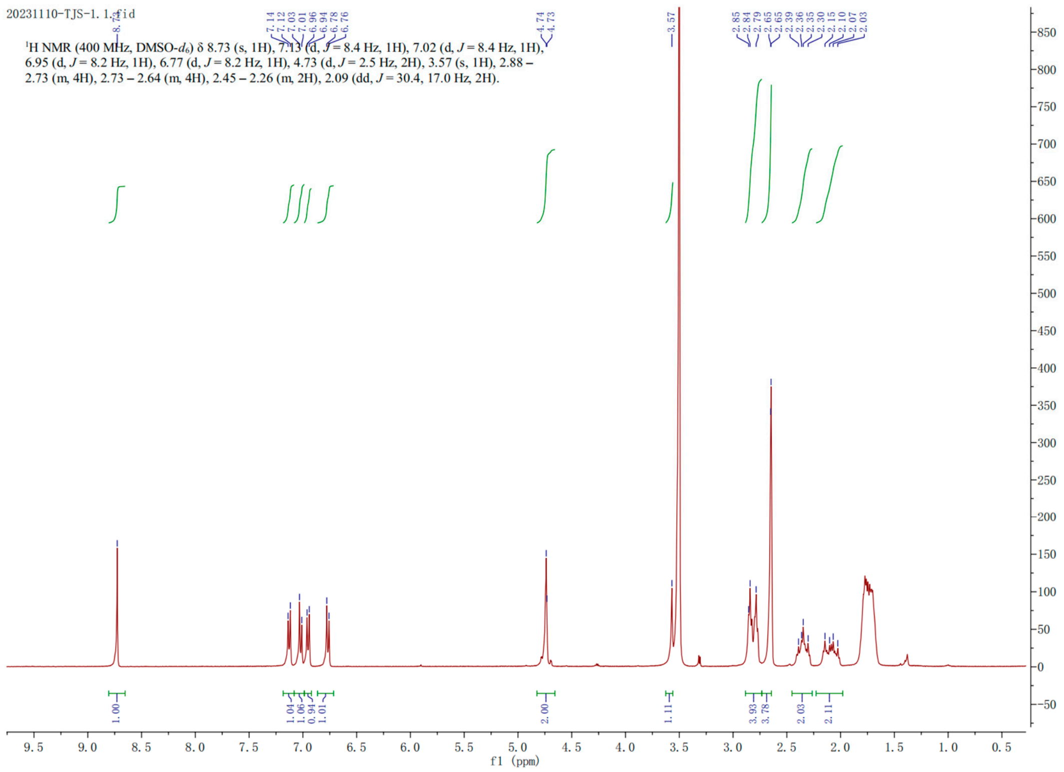

Figure S3 ^{1}H NMR of *R*-**P** (DMSO-d$_6$)

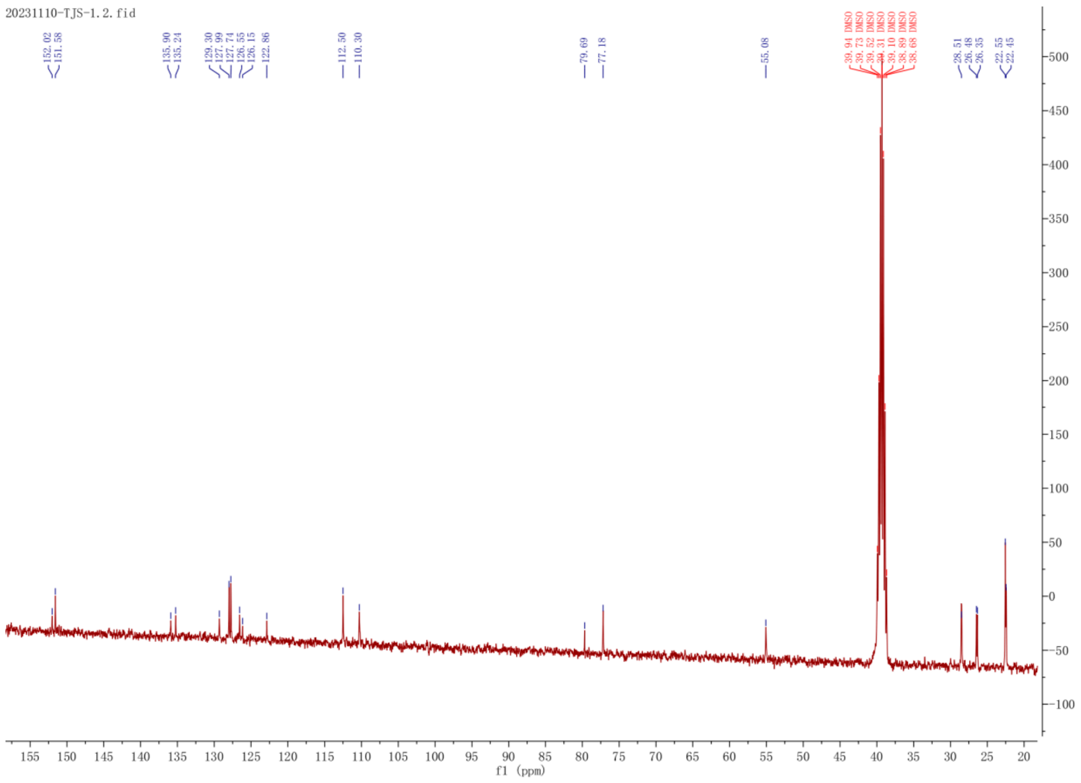

Figure S4 ^{13}C NMR of *R*-**P** (DMSO-d$_6$)

4. ^{1}H NMR, ^{13}C NMR of *R*-β-D-**1**

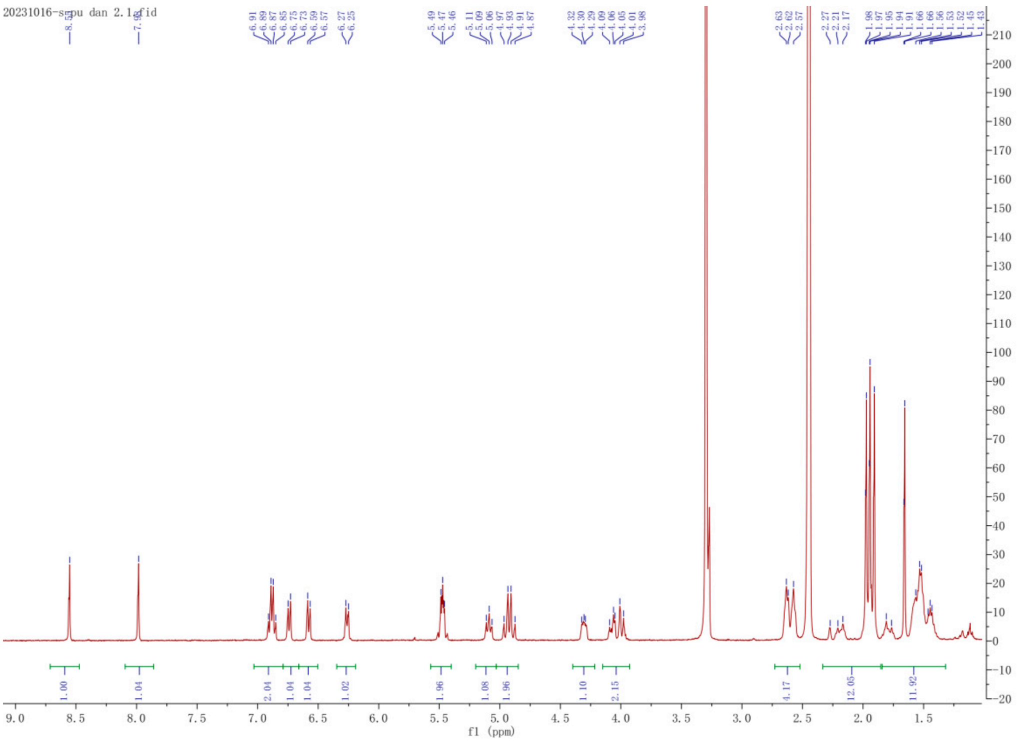

Figure S5 ^{1}H NMR of *R*-β-D-**1** (DMSO-d_6)

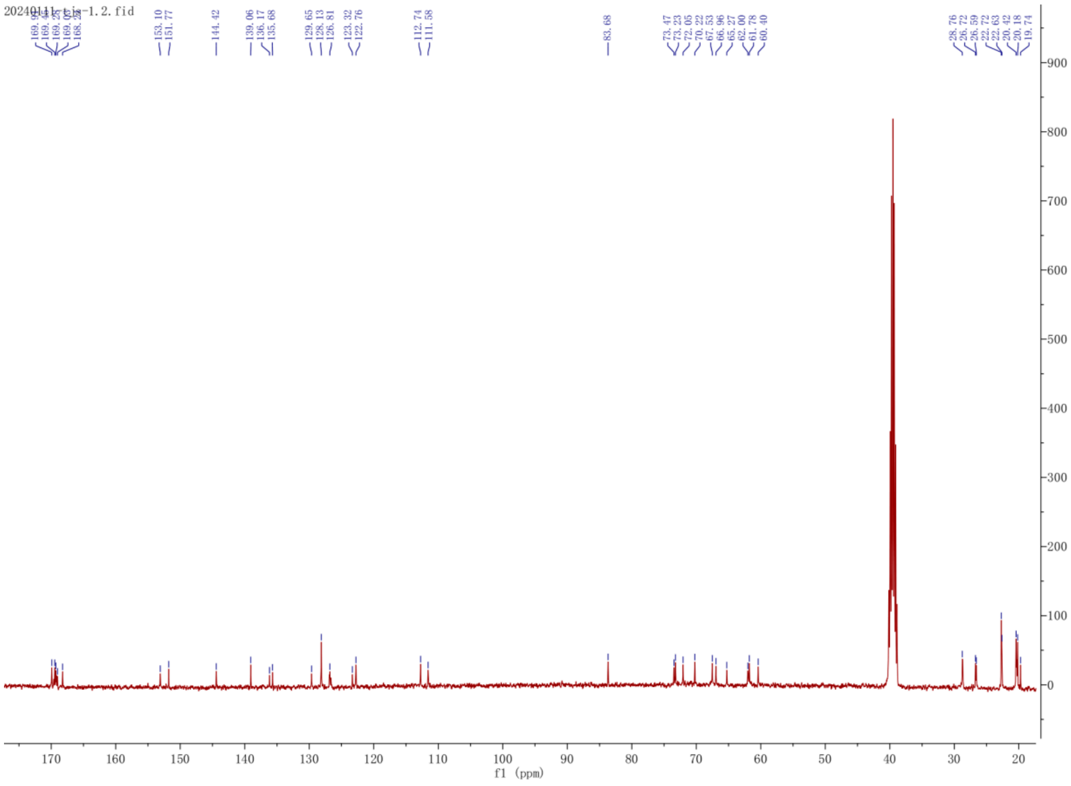

Figure S6 ^{13}C NMR of *R*-β-D-**1** (DMSO-d_6)

5. MS plot of *R*-β-D-**1**

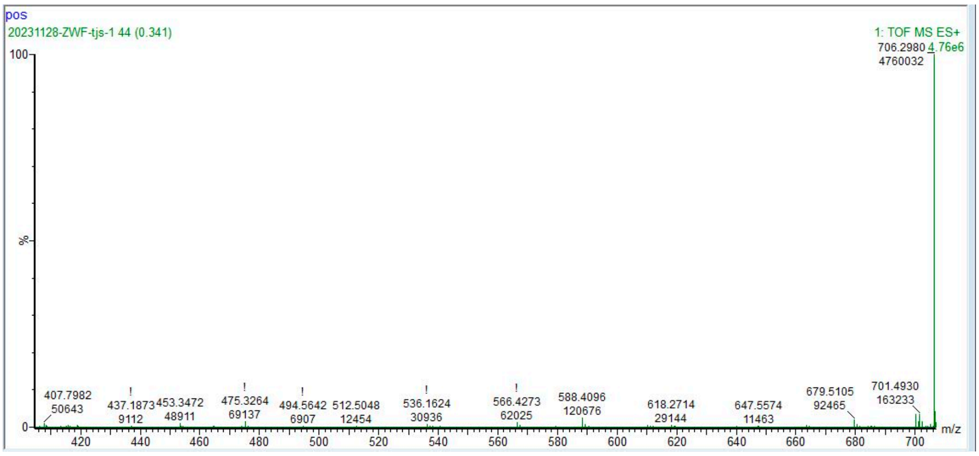

Figure S7 mass spectrometry of *R*-β-D-**1**

6.Histogram of *R*-β-D-**1** with Bi^{3+} in the presence of different anions

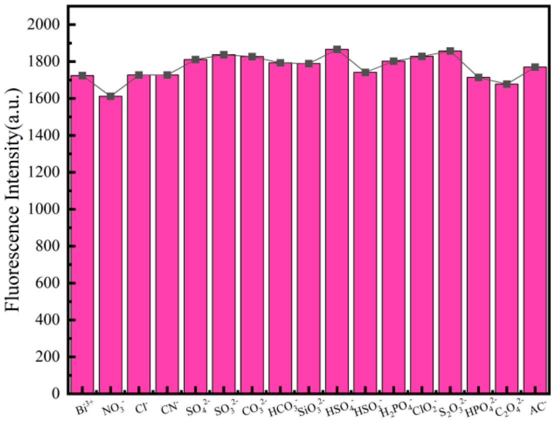

Figure S8 Fluorescence histogram of *R*-β-D-**1** versus Bi^{3+} in the presence of different anions

7.Preparation of fluorescence test samples

7 mg of probe *R*-β-D-**1** in a 10 mL volumetric flask, add chromatographic methanol to dissolve and quantify to 10 mL, at this time, the concentration of the test masterbatch is 0.001 M. Next, the masterbatch will be diluted to 2.0×10^{-5} M, and taken at any time when needed.

Commonly used ion configuration ($BaCl_2$, $MnCl_2$, CuCl, $CaCl_2$, KCl, $CoCl_3$, $CrCl_3$, $ZnCl_2$, $AlCl_3$, $MgCl_2$, $PbCl_2$, AgCl, $CdCl_2$, $GdCl_3$, LiCl, NaCl, NH_4Cl, $NiCl_2$, $BiCl_3$ configured into a concentration of 0.1 M)(Methanol solution, need to be ready to use, room temperature 25°C, take 2mL test solution added to 3.5mL high transparent quartz fluorescence cuvette, add 2μL of ions to be tested, fluorescence test).

Commonly used anionic formulations (KNO_3, NaCl, NaCN, Na_2SO_4, Na_2SO_3, Na_2CO_3, $NaHCO_3$, Na_2SiO_3, $NaHSO_4$, $NaHSO_3$, NaH_2PO_4, $NaClO_2$, $Na_2S_2O_3$, $NaHPO_4$, $Na_2C_2O_4$, and CH_3COONa configured into a concentration of 0.1 M) (water solution, the solution should be ready to use, room temperature 25℃, take 2mL of the test solution and add it into a 3.5mL high transparent quartz fluorescence cuvette, add 2 μL Bi^{3+} ions, then add 2 μL of the anionic to be tested for the fluorescence test).

8.Method of calculating charge density difference (CDD)

Ab initio calculations were performed with the periodic density functional theory (DFT) code Vienna ab initio simulation package (VASP) [1,2]. The exchange and correlation energy was calculated within the generalized gradient approximation (GGA) using the Perdew−Burke−Ernzerhof (PBE) functional [3,4]. To include van der Waals forces, we added the D3 correction as implemented by Grimme et al [5,6]. The electron-core interaction was described with the projector augmented wave (PAW) method [7]. The electronic wave functions were expanded using a plane wave basis set with an energy cutoff of 400 eV. Gamma type k-point meshes of 1 × 1 × 1 were used for electronic structure analysis [8-12].

9.SEM plots of the sensor *R*-β-D-**1** with Bi^{3+} in methanol

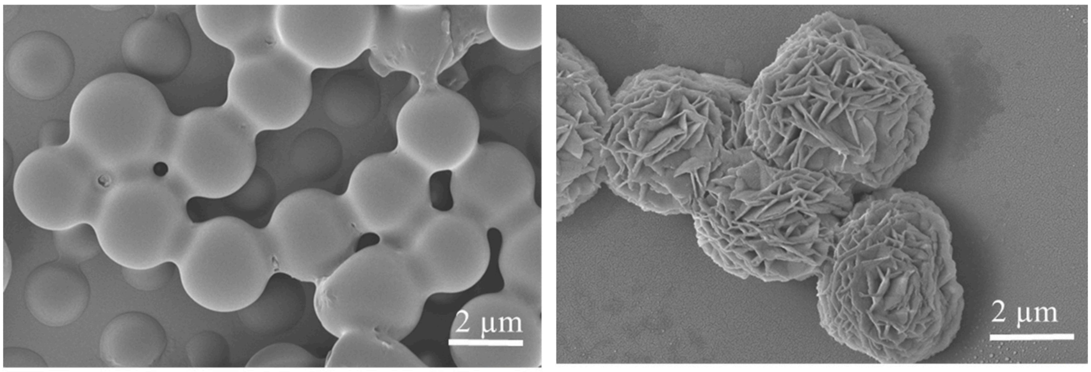

a b

Figure S9 a SEM image of *R*-β-D-**1** in methanol solvent, b is the SEM morphology change of *R*-β-D-**1** with the addition of Bi^{3+}.

References

1. Kresse, G.; Furthmüller, J., Efficiency of ab-initio total energy calculations for metals and semiconductors using a plane-wave basis set. *Comput. Mater. Sci.* **1996,** *6* (1), 15-50.

2. Kresse, G.; Furthmüller, J., Efficient iterative schemes for ab initio total-energy calculations using a plane-wave basis set. *Phys. Rev. B* **1996,** *54* (16), 11169-11186.

3. Perdew, J. P.; Burke, K.; Ernzerhof, M., Generalized Gradient Approximation Made Simple. *Phys. Rev. Lett.* **1996,** *77* (18), 3865-3868.

4. Perdew, J. P.; Ernzerhof, M.; Burke, K., Rationale for mixing exact exchange with density functional approximations. *The Journal of Chemical Physics* **1996,** *105* (22), 9982-9985.
5. Grimme, S.; Antony, J.; Ehrlich, S.; Krieg, H., A consistent and accurate ab initio parametrization of density functional dispersion correction (DFT-D) for the 94 elements H-Pu. *The Journal of Chemical Physics* **2010,** *132* (15), 154104.
6. Grimme, S.; Ehrlich, S.; Goerigk, L., Effect of the damping function in dispersion corrected density functional theory. *J. Comput. Chem.* **2011,** *32* (7), 1456-1465.
7. Blöchl, P. E., Projector augmented-wave method. *Phys. Rev. B* **1994,** *50* (24), 17953-17979.
8. Heyden, A.; Bell, A. T.; Keil, F. J., Efficient methods for finding transition states in chemical reactions: Comparison of improved dimer method and partitioned rational function optimization method. *The Journal of Chemical Physics* **2005,** *123* (22), 224101.
9. Wang, V.; Xu, N.; Liu, J.-C.; Tang, G.; Geng, W.-T., VASPKIT: A user-friendly interface facilitating high-throughput computing and analysis using VASP code. *Comput. Phys. Commun.* **2021,** *267*, 108033.
10. Hjorth Larsen, A.; Jorgen Mortensen, J.; Blomqvist, J.; Castelli, I. E.; Christensen, R.; Dulak, M.; Friis, J.; Groves, M. N.; Hammer, B.; Hargus, C.; Hermes, E. D.; Jennings, P. C.; Bjerre Jensen, P.; Kermode, J.; Kitchin, J. R.; Leonhard Kolsbjerg, E.; Kubal, J.; Kaasbjerg, K.; Lysgaard, S.; Bergmann Maronsson, J.; Maxson, T.; Olsen, T.; Pastewka, L.; Peterson, A.; Rostgaard, C.; Schiotz, J.; Schutt, O.; Strange, M.; Thygesen, K. S.; Vegge, T.; Vilhelmsen, L.; Walter, M.; Zeng, Z.; Jacobsen, K. W., The atomic simulation environment-a Python library for working with atoms. *J Phys Condens Matter* **2017,** *29* (27), 273002.
11. Momma, K.; Izumi, F., VESTA: a three-dimensional visualization system for electronic and structural analysis. *J. Appl. Crystallogr.* **2008,** *41* (3), 653-658.
12. Peterson, A. A.; Abild-Pedersen, F.; Studt, F.; Rossmeisl, J.; Nørskov, J. K., How copper catalyzes the electroreduction of carbon dioxide into hydrocarbon fuels. *Energy Environ. Sci.* **2010,** *3* (9), 1311-1315.
